# Exploring the Use of Compound-Induced Transcriptomic Data Generated From Cell Lines to Predict Compound Activity Toward Molecular Targets

**DOI:** 10.3389/fchem.2020.00296

**Published:** 2020-04-23

**Authors:** Benoît Baillif, Joerg Wichard, Oscar Méndez-Lucio, David Rouquié

**Affiliations:** ^1^Bayer SAS, Bayer CropScience, Sophia Antipolis, France; ^2^Department of Genetic Toxicology, Bayer AG, Berlin, Germany; ^3^Bloomoon, Villeurbanne, France

**Keywords:** target prediction, compound-induced transcriptomic data, QSAR, machine learning, cellular context

## Abstract

Pharmaceutical or phytopharmaceutical molecules rely on the interaction with one or more specific molecular targets to induce their anticipated biological responses. Nonetheless, these compounds are also prone to interact with many other non-intended biological targets, also known as off-targets. Unfortunately, off-target identification is difficult and expensive. Consequently, QSAR models predicting the activity on a target have gained importance in drug discovery or in the de-risking of chemicals. However, a restricted number of targets are well characterized and hold enough data to build such *in silico* models. A good alternative to individual target evaluations is to use integrative evaluations such as transcriptomics obtained from compound-induced gene expression measurements derived from cell cultures. The advantage of these particular experiments is to capture the consequences of the interaction of compounds on many possible molecular targets and biological pathways, without having any constraints concerning the chemical space. In this work, we assessed the value of a large public dataset of compound-induced transcriptomic data, to predict compound activity on a selection of 69 molecular targets. We compared such descriptors with other QSAR descriptors, namely the Morgan fingerprints (similar to extended-connectivity fingerprints). Depending on the target, active compounds could show similar signatures in one or multiple cell lines, whether these active compounds shared similar or different chemical structures. Random forest models using gene expression signatures were able to perform similarly or better than counterpart models built with Morgan fingerprints for 25% of the target prediction tasks. These performances occurred mostly using signatures produced in cell lines showing similar signatures for active compounds toward the considered target. We show that compound-induced transcriptomic data could represent a great opportunity for target prediction, allowing to overcome the chemical space limitation of QSAR models.

## Introduction

Biologically active molecules rely on the interaction with one or more molecular targets (Hughes et al., 2000). In the context of hit discovery both in pharmaceutical or in phytopharmaceutical industries, a major objective is to be able to screen molecule candidates for their activity toward a target of interest, and assessing compound activity toward off-targets, that can cause adverse effects *in vivo* (Rouquié et al., 2015). Testing activity of every candidate on a battery of targets represent a complex task that requires major R&D costs. A potential solution to predict candidate's activity with a lower cost is to perform computational methods using more general measured or calculated descriptors (Chen et al., 2016; Vamathevan et al., 2019).

A commonly used technique is to compute descriptors from chemical structures, like the extended-connectivity fingerprints (ECFPs) and use them for prediction, relying on the quantitative structure-activity relationship (QSAR) principle, i.e., molecules sharing a similar structure may share a similar activity profile (Rogers and Hahn, 2010; Cherkasov et al., 2014). However, such molecule descriptors show limitations: they do not perform well for all target prediction tasks depending on the quantity and quality of available activity data, prediction is limited to the applicability domain (depending on the training set used), and a small change in chemical structure can lead to a large change in biological response (activity cliffs) (Cruz-Monteagudo et al., 2014).

Additional descriptors have been proposed to circumvent such QSAR drawbacks, such as measurements from large scale biological assays (Petrone et al., 2012; Laufkötter et al., 2019). Results from high throughput screening (HTS) assays, such as bioactivity experiments, can be used as fingerprints (HTSFPs) in predictive models for specific targets. Petrone et al. (2012) showed that models using HTSFPs were outperforming models using ECFPs for certain targets, and that HTSFP models' predictions were covering a large structural diversity. The main limiting factor of such models is the sparsity of available activity data. Besides bioactivity data, more integrative large-scale biological measurements, like transcriptomics or cell morphology readouts can be used for target prediction (Aliper et al., 2016; Pabon et al., 2018; Scheeder et al., 2018; Simm et al., 2018; Hofmarcher et al., 2019; Kuthuru et al., 2019; Lapins and Spjuth, 2019).

Compound-induced gene expression data are gathered from biological experiments reflecting how the compound acted on one or multiple targets in a specific biological context. Cancer cell lines, being easily cultured, are a commonly used model to generate gene expression data. Hughes et al. (2000) proved that enough data allows to use pattern-matching algorithms to study similarity between signatures coming from drug induction (Hughes et al., 2000). Lamb et al. (2006) invented the concept of Connectivity Map (CMAP), creating relationships between small molecules, genes and diseases (Lamb et al., 2006). Since then, transcriptomics data have been shown to be useful to identify new molecules with biological activity (Hieronymus et al., 2006; Wei et al., 2006). Recently, a large public CMAP L1000 dataset was released representing more than 300,000 Gene Expression Signatures (GESs) of cell line responses to so-called perturbagens (Subramanian et al., 2017). GESs were produced for more than 20,000 compounds in 80 human cancer cell lines, tested at various concentration and exposition time. The large scale of this dataset allows the use of GESs in machine learning models for target prediction or drug repurposing (Lee et al., 2016; De Wolf et al., 2018).

In the current work, we investigated whether we could predict compound activity toward a larger number of molecular targets based on their GESs extracted from the CMAP L1000 dataset. In addition, we were interested to reveal how machine learning models using GESs perform compared to models using more traditional QSAR descriptors, such as the Morgan fingerprints.

We show that random forest models built using compound-induced GES were able to effectively predict targets, especially if they were produced from a cell line showing similar GESs between active compounds on the evaluated target. For 25% of the target prediction tasks, GESs models had similar or higher performances than models using Morgan fingerprints, offering an opportunity to escape from the chemical space limitation associated with QSAR approaches.

## Materials and Methods

### Gene Expression Signatures (GESs) Acquisition

The CMAP L1000 dataset was obtained from two GEO repositories: GSE92742, corresponding to the first phase of L1000 (pilot, 2012–2015) and GSE70138, which is the second phase (production, on-going). GESs generation was described by Subramanian et al. (2017).

For this study, we only used Level 5 GESs meaning that each GES is represented by an instance, that is a combination of a perturbagen (chemical or gene deletion), cell line, concentration and time point, and is composed by the plate-normalized expression z-scores of the whole genome, inferred from 978 landmark genes (measured gene that can be used for whole transcriptome inference). We focused on landmark signatures of compound perturbagens, which comprises 333,273 GESs for 21,300 unique compounds. GESs obtained in the exact same condition were averaged, to have one signature per condition.

**Figure 1:**
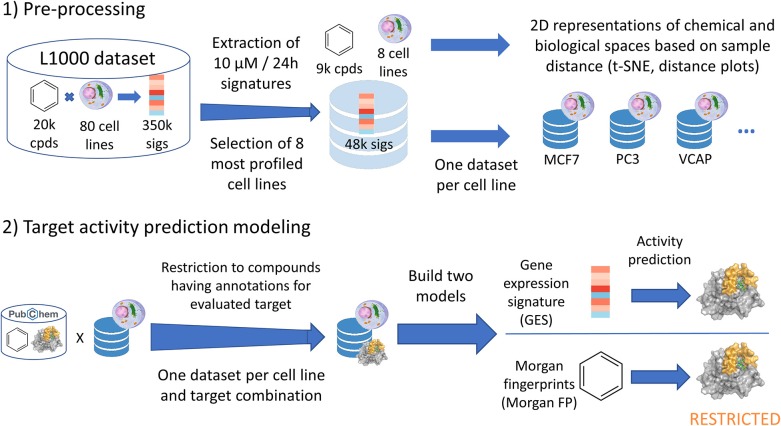
Data analysis pipeline performed in current work. Starting from the CMAP L1000 dataset, signatures produced at 10 μM and 24 h from 8 cell lines were extracted and used in t-SNE and distance plots. One dataset was built per cell line (GES and corresponding compound structure), and each of these datasets were restricted to compounds having known annotations (active or inactive) for the evaluated target. For each target—cell line dataset, a first model was built using the gene expression signatures (GES model). Alongside, a second counterpart model was built using the Morgan fingerprints of compounds whose signatures were used in the first model (Morgan FP model).

Among all obtained GESs, the ones generated at a 10 μM and 24 h time point were selected (as shown in Figure 1), as this condition was the most represented in the dataset and facilitate the comparison of results. GESs from the 8 most profiled cell lines were used; cell line and number of GESs are presented in Table 1. Also, only GESs generated by compounds with known structure were selected. In total, the working dataset contains 39,544 GESs obtained from 9,035 compounds.

**Table 1:** The 8 core cell lines used in this work, with their corresponding number of GESs for compounds with known structure tested at 10 μM/24 h.

**Cell line**	**Primary site**	**Subtype**	**Number of 10 μM−24 h signatures**
A375	Skin	Malignant melanoma	3,525
A549	Lung	Non small cell lung cancer| carcinoma	5,267
HA1E	Kidney	Normal kidney	3,646
HCC515	Lung	Carcinoma	1,932
HT29	Large intestine	Colorectal adenocarcinoma	3,192
MCF7	Breast	Adenocarcinoma	7,546
PC3	Prostate	Adenocarcinoma	8,071
VCAP	Prostate	Carcinoma	6,365

### Activity Data Acquisition

Annotations about activity or inactivity was retrieved from the PubChem BioAssay database, using available CIDs documented in the L1000 signature metadata, excepted for TUBB actives, that were extracted from the Drug Repurposing Hub of the LINCS (Wang et al., 2014; Corsello et al., 2017). Activity data were compiled in a binary activity matrix (1 for active, 0 for inactive, empty if unknown). At least one annotation among 1,388 targets was found for 7,804 of the 9,035 compounds (512,406 annotations were found, representing 4.8% of the full activity matrix).

### Representation of Chemical and Biological Spaces

For each compound, binary Morgan fingerprints were computed. The Morgan fingerprints were employed as input of a t-SNE (t-distributed stochastic neighbor embedding) algorithm (using the sklearn implementation) using Dice distance as metric, to reduce the data to a two-dimensional output that can be plotted to represent the chemical space (Van Der Maaten and Hinton, 2008; Pedregosa et al., 2011). Information of the number of targets per compound was included as color-code using a blue gradient in the plot.

The whole extracted 10 μM/24 h signature dataset was used as input for a second t-SNE using the cosine distance metric, representing the overall biological (response) space wherein each cell line was color-coded in the plot. For every cell line, a t-SNE using the cosine distance metric was performed using all GESs profiled in the cell line, generating 2D biological space.

### Machine Learning Modeling

Targets for which we know at least 50 active compounds (representing between 1 and 63% of active per target) were selected for machine learning modeling in order to have a minimum number of actives in test sets to evaluate the model performances, and for computational time purposes. Complete information on the number of active and inactive compounds for these selected targets is listed in Table 2.

**Table 2:** Molecular targets used in this work, with number of active and inactive compounds in total, and in each cell line GES dataset.

**Gene name**	**Total inactive**	**Total active**	**Description**	**A375**		**A549**		**HA1E**		**HCC515**		**HT29**		**MCF7**		**PC3**		**VCAP**	
				**Inactive**	**Active**	**Inactive**	**Active**	**Inactive**	**Active**	**Inactive**	**Active**	**Inactive**	**Active**	**Inactive**	**Active**	**Inactive**	**Active**	**Inactive**	**Active**
ABCB1	801	96	ATP binding cassette subfamily B member 1 [HGNC:40]	331	42	545	60	424	50	211	32	317	32	770	95	772	95	608	68
ABHD5	2,458	57	Abhydrolase domain containing 5 [HGNC:21396]	908	22	1,533	47	-	-	-	-	-	-	2,030	57	2,045	57	1,730	52
ALOX15	1,136	101	Arachidonate 15-lipoxygenase [HGNC:433]	508	27	764	92	808	52	496	33	498	27	1,062	97	1,066	96	804	91
AR	1,085	103	Androgen receptor [HGNC:644]	580	36	639	64	757	62	374	36	572	37	1,038	94	1,036	93	682	69
ATAD5	2,213	97	Atpase family, AAA domain containing 5 [HGNC:25,752]	1,007	43	1,466	70	1288	60	638	41	922	42	2,087	90	2,090	88	1,629	72
ATXN2	1,897	143	Ataxin 2 [HGNC:10555]	695	36	1,139	104	950	50	501	41	652	41	1556	123	1,558	129	1,243	102
BAZ2B	1,252	143	Bromodomain adjacent to zinc finger domain 2B [HGNC:963]	516	63	873	101	653	76	319	37	470	56	1,199	135	1,197	136	974	112
BRCA1	3,008	160	BRCA1, dna repair associated [HGNC:1100]	1,117	67	1945	116	1,519	63	800	33	1,014	50	2,537	148	2,549	150	2,134	131
CBX1	1,999	80	Chromobox 1 [HGNC:1551]	899	41	1,412	64	1,120	61	532	38	809	40	1,899	75	1,904	76	1,576	67
CHRM1	2,433	86	Cholinergic receptor muscarinic 1 [HGNC:1950]	906	49	1,544	62	1,055	59	460	39	791	48	2,036	84	2051	83	1,739	65
CHRM4	2,476	70	Cholinergic receptor muscarinic 4 [HGNC:1953]	908	45	1,552	54	1,057	55	460	40	793	45	2,049	68	2,064	67	1,751	54
CHRM5	2,478	62	Cholinergic receptor muscarinic 5 [HGNC:1954]	908	41	1,553	47	1,057	49	461	36	793	39	2,050	62	2,065	61	1,751	50
CYP1A2	307	526	Cytochrome P450 family 1 subfamily A member 2 [HGNC:2596]	145	265	183	399	219	428	136	277	144	261	299	505	294	505	183	410
CYP2C19	717	276	Cytochrome P450 family 2 subfamily C member 19 [HGNC:2621]	329	151	514	213	486	228	289	138	324	148	688	271	684	272	524	225
CYP2C9	708	270	Cytochrome P450 family 2 subfamily C member 9 [HGNC:2623]	310	157	510	197	476	222	285	126	305	154	679	264	674	263	517	207
CYP3A4	1,153	164	Cytochrome P450 family 3 subfamily A member 4 [HGNC:2637]	472	113	780	104	847	133	561	64	467	110	1,070	160	1069	161	802	118
DRD1	1,843	99	Dopamine receptor D1 [HGNC:3020]	807	54	1,295	71	1028	78	526	55	725	54	1,762	91	1,762	91	1,450	71
DRD2	2,262	95	Dopamine receptor D2 [HGNC:3023]	769	58	1,371	73	956	84	474	55	683	57	1,858	93	1873	93	1,541	74
DRD3	2,446	142	Dopamine receptor D3 [HGNC:3024]	877	76	1,432	110	1,129	114	551	78	823	75	2,004	139	2,017	139	1,569	111
EPAS1	2,443	70	Endothelial PAS domain protein 1 [HGNC:3374]	–	–	1,524	52	–	–	–	–	–	–	2,021	64	2,033	67	1,723	57
FEN1	2,100	53	Flap structure-specific endonuclease 1 [HGNC:3650]	961	23	1,496	29	1,213	28	–	–	866	21	1,990	46	1,999	46	1,669	32
GFER	1,589	89	Growth factor, augmenter of liver regeneration [HGNC:4236]	679	37	1,153	59	813	46	363	21	600	29	1,519	81	1,519	81	1,294	70
GLS	2,989	66	Glutaminase [HGNC:4331]	1,240	22	1,878	46	1,515	31	–	–	–	–	2,560	58	2,574	59	2,072	53
GMNN	2,079	161	Geminin, DNA replication inhibitor [HGNC:17493]	969	67	1,392	121	1,224	72	569	43	884	63	1,972	146	1,974	153	1,552	128
HPGD	1,464	92	15-Hydroxyprostaglandin dehydrogenase [HGNC:5154]	575	38	1,000	74	962	62	618	39	567	37	1363	89	1,363	86	1,060	71
HSD17B10	1,211	107	Hydroxysteroid 17-beta dehydrogenase 10 [HGNC:4800]	516	48	827	84	858	81	548	47	506	48	1,134	99	1,135	95	866	81
HSP90AA1	666	56	Heat shock protein 90 alpha family class A member 1 [HGNC:5253]	295	25	453	39	419	38	–	–	288	25	640	50	637	50	502	40
HSPB1	876	76	Heat shock protein family B (small) member 1 [HGNC:5246]	461	40	522	45	600	53	304	26	454	41	837	72	837	71	558	47
HTR1A	412	60	5-Hydroxytryptamine receptor 1A [HGNC:5286]	186	34	279	49	232	55	122	37	180	34	401	58	400	58	315	50
IL1B	1,773	206	Interleukin 1 beta [HGNC:5992]	589	54	1,005	165	768	78	382	54	541	54	1,385	190	1,391	196	1,122	163
JAK2	895	80	Janus kinase 2 [HGNC:6192]	378	39	663	58	478	43	248	29	364	40	867	71	867	74	723	55
JUN	842	97	Jun proto-oncogene, AP-1 transcription factor subunit [HGNC:6204]	442	49	491	67	570	72	279	41	435	49	801	91	799	91	523	70
KCNH2	363	190	Potassium voltage-gated channel subfamily H member 2 [HGNC:6251]	174	119	212	136	250	161	128	104	173	119	331	183	329	184	228	139
KDM4A	1,607	192	Lysine demethylase 4A [HGNC:22978]	693	76	1,130	125	834	87	379	42	603	69	1,529	173	1,536	175	1,286	140
KDM4E	1,389	124	Lysine demethylase 4E [HGNC:37098]	543	43	999	88	880	70	547	40	530	42	1,320	109	1,321	110	1,057	95
MITF	3,626	132	Melanogenesis associated transcription factor [HGNC:7105]	1,170	42	2,238	91	1,562	51	858	42	1,083	37	2,832	116	2,871	120	2,460	93
MLLT3	14,566	101	MLLT3, super elongation complex subunit [HGNC:7136]	–	–	2,244	26	–	–	–	–	–	–	3,002	33	3,461	50	3,095	46
MPHOSPH8	506	52	M-Phase phosphoprotein 8 [HGNC:29810]	278	21	365	39	403	41	253	29	278	21	490	48	485	50	382	43
MYC	2,069	121	MYC proto-oncogene, bHLH transcription factor [HGNC:7553]	–	–	1,067	113	–	–	–	–	–	–	1,230	114	1,249	117	1,151	115
NFE2L2	2,850	226	Nuclear factor, erythroid 2 like 2 [HGNC:7782]	1,142	94	1,816	148	1,355	153	620	83	1,013	95	2,425	204	2,439	204	2,023	152
NFKB1	2,875	107	Nuclear factor kappa B subunit 1 [HGNC:7794]	730	23	1,608	91	1,237	37	814	29	716	22	1,978	100	2,000	101	1,742	94
NOD1	1,056	51	Nucleotide binding oligomerization domain containing 1 [HGNC:16390]	–	–	754	43	–	–	–	–	409	21	1,010	47	1,010	49	844	40
NOD2	2,578	59	Nucleotide binding oligomerization domain containing 2 [HGNC:5331]	952	23	1,629	53	1,124	21	–	–	836	23	2,152	57	2,165	59	1,837	48
NPSR1	1,007	55	Neuropeptide S receptor 1 [HGNC:23631]	–	–	712	44	554	21	–	–	–	–	959	52	956	54	777	50
NR3C1	925	54	Nuclear receptor subfamily 3 group C member 1 [HGNC:7978]	–	–	636	38	692	34	451	25	–	–	896	54	890	54	659	47
NR5A1	419	69	Nuclear receptor subfamily 5 group A member 1 [HGNC:7983]	190	22	285	50	239	29	–	–	–	–	408	60	407	64	322	50
OPRK1	1,122	51	Opioid receptor kappa 1 [HGNC:8154]	455	29	805	41	564	34	–	–	428	29	1,068	51	1,070	50	895	41
PIP4K2A	1,898	88	Phosphatidylinositol-5-phosphate 4-kinase type 2 alpha [HGNC:8997]	609	31	1,082	66	812	34	–	–	566	23	1,486	84	1,501	83	1,206	72
PLA2G7	1,907	57	Phospholipase A2 group VII [HGNC:9040]	828	27	1,367	37	995	37	–	–	737	28	1,825	52	1,826	51	1,534	37
PLK1	1,935	108	Polo like kinase 1 [HGNC:9077]	631	45	1,125	82	836	39	431	23	590	36	1,531	100	1,542	102	1,241	88
POLB	1,166	53	DNA polymerase beta [HGNC:9174]	480	22	850	37	646	30	–	–	462	21	1,113	50	1,113	50	932	39
POLH	2,202	70	DNA polymerase eta [HGNC:9181]	858	26	1,342	44	1,015	37	–	–	749	23	1,827	61	1,838	64	1,532	48
POLI	1,726	79	DNA polymerase iota [HGNC:9182]	775	29	1,210	52	945	43	448	24	689	27	1,629	71	1,637	72	1,359	55
POLK	2,895	79	DNA polymerase kappa [HGNC:9183]	1,248	30	2,048	49	1,799	43	986	27	1,154	26	2,713	69	2,722	70	2,225	54
PRMT1	2,886	80	Protein arginine methyltransferase 1 [HGNC:5187]	1,114	28	1,704	59	1,073	23	–	–	–	–	2,394	74	2,415	74	2,081	68
RAD52	14,593	132	RAD52 homolog, DNA repair protein [HGNC:9824]	–	–	2,291	25	–	–	–	–	–	–	3,043	40	3,496	54	3,121	49
SIRT5	14,103	141	Sirtuin 5 [HGNC:14933]	–	–	2,086	30	–	–	–	–	–	–	2,769	40	3,211	44	2,844	42
SLC6A3	1,006	94	Solute carrier family 6 member 3 [HGNC:11049]	461	49	773	71	584	72	252	54	453	49	976	91	973	90	823	73
SMN2	1,633	53	Survival of motor neuron 2, centromeric [HGNC:11118]	–	–	1,136	44	1,059	28	–	–	–	–	1,520	49	1,521	49	1,209	45
STK33	3,358	423	Serine/threonine kinase 33 [HGNC:14568]	1,127	101	2,077	304	1,458	163	776	131	1,034	102	2,660	329	2,663	360	2,268	328
TARDBP	1,802	60	TAR DNA binding protein [HGNC:11571]	–	–	1,044	50	748	25	–	–	–	–	1,409	58	1,418	59	1,156	52
TNFRSF10B	2,429	80	TNF receptor superfamily member 10b [HGNC:11905]	–	–	1,510	66	1,056	26	–	–	786	27	2,008	73	2,021	75	1,718	58
TP53	2,310	198	Tumor protein p53 [HGNC:11998]	974	97	1,554	137	1,380	130	737	84	891	98	2,172	179	2,174	181	1,714	137
TSHR	2,259	70	Thyroid stimulating hormone receptor [HGNC:12373]	968	25	1,579	58	1,317	43	727	35	–	–	2,133	68	2,131	67	1,739	64
TUBB	697	51	Tubulin beta class I [HGNC:20778]	–	–	503	32	373	32	–	–	–	–	692	48	693	49	563	32
USP1	2,356	64	Ubiquitin specific peptidase 1 [HGNC:12607]	877	30	1,425	46	1,260	45	697	29	833	30	1,972	55	1,985	58	1,557	44
VDR	2,696	140	Vitamin D receptor [HGNC:12679]	1,161	44	1,901	101	1,673	80	915	50	1,076	43	2,530	127	2,536	128	2,059	107
YES1	138	101	YES proto-oncogene 1, Src family tyrosine kinase [HGNC:12841]	106	89	99	86	117	89	77	76	106	89	132	97	131	99	90	67

Subsequently, for each cell line GES dataset, we created a target—cell line GES dataset, restricting to compounds for which target activity was known as shown in Figure 1 (this step caused the number of possible models to drop from 1,104 to 990). Datasets for each target prediction task were split into a training set (67% of the data) and a test set (remaining 33% of the data). Two models for target activity prediction were trained using each subset: a first model used the 978-landmark GES as input (referred as GES models), and the second one used the Morgan fingerprints of corresponding compounds to fairly compare model performances (referred as Morgan FP models). Models were trained using random forest classifiers (Breiman, 2001).

The training was performed using a 4-fold cross-validation on training set to tune the maximum depth of tree, before assessing prediction performances on the test set. The number of trees per model was set to 200. Models were built in Python 2.7 using the sklearn package: to account for unbalanced dataset, the “class_weight” parameter was set to “balanced_subsample,” to increase the weight of the under-represented class samples when training the trees (Pedregosa et al., 2011). A first step of feature selection was performed using an initial random forest classifier, computing the feature importance (Breiman, 2001). Sum of importance of all feature was 1, with each feature importance between 0 (non-important) and 1 (important). This step was performed 5 times, feature importance was averaged by feature, and only the 20 most important features were selected to be loaded into a final random forest model. This whole modeling pipeline, from train-test split to final model was performed 10 times per task, to account for variable performances depending on the dataset split.

Models were evaluated by counting the numbers of true positive (TP), true negative (TN), false positive (FP) and false negative (FN). These parameters were combined in the following metrics in order to compare model performances:

$$\begin{array}{l} •Sensitivity=TP/\left(TP+\;FN\right)\\ •Specificity=TN/\left(TN+\;FP\right)\\ •\text{Balanced accuracy }\left(\text{BA}\right):BA=\left(Sensitivity+\;Specificity\right)/2\\ •\text{Matthews correlation coefficient }\left(\text{MCC}\right):\\ \text{MCC}=\frac{\text{T}{\text{P}}^{*}\text{TN}-\text{F}{\text{P}}^{*}\text{FN}}{\sqrt{\left(\text{TP}+\text{FP}\right)\left(\text{TP}+\text{FN}\right)\left(\text{TN}+\text{FP}\right)\left(\text{TN}+\text{FN }\right)}} \end{array}$$

Balanced accuracy allows for a fair evaluation of model performances when using unbalanced datasets, by averaging accuracy for each class (here active and inactives).

### Quadrant Plots

Between each possible pair of compounds active on the same target and in each cell line, Dice distance between Morgan fingerprints, and cosine distance between GESs in given cell line were computed. These 2 distances were plotted in a 2D plot (referred as distance plot), Dice distance on X-axis and cosine distance on Y-axis. These plots were theoretically split in 4 quadrants.

Quadrant I in the top-right corner contains active compound pairs having different structures (Morgan fingerprints Dice distance >0.5) and presenting different GESs (cosine distance >0.5); quadrant II in top-left corner contains active compound pairs having similar structures (Morgan fingerprints Dice distance <0.5) and presenting different GESs (cosine distance >0.5); quadrant III in bottom-left corner contains active compound pairs having similar structures (Morgan fingerprints Dice distance <0.5) and presenting similar GESs (cosine distance <0.5); quadrant IV in bottom-right corner contains active compound pairs having different structures (Morgan fingerprints Dice distance >0.5) and presenting similar GESs (cosine distance <0.5). Number of active compound pairs in each quadrant were counted for each distance plot. Similar calculations were made using not only active compounds, but all compounds having an annotation (active or inactive) for considered target and profiled in the same cell line.

## Results

In the present work, we investigated the link between compound structure information (*n* = 9,035) and their corresponding induced biological responses captured by GESs (*n* = 39,544) in human tumor cell lines and evaluated the potential of machine learning approaches to infer about molecular targets involved in the compound bioactivity. In addition, we compared these machine learning models using GESs with counterpart models using Morgan fingerprints.

### Exploration of Chemical and Biological Spaces

**Figure 2:**
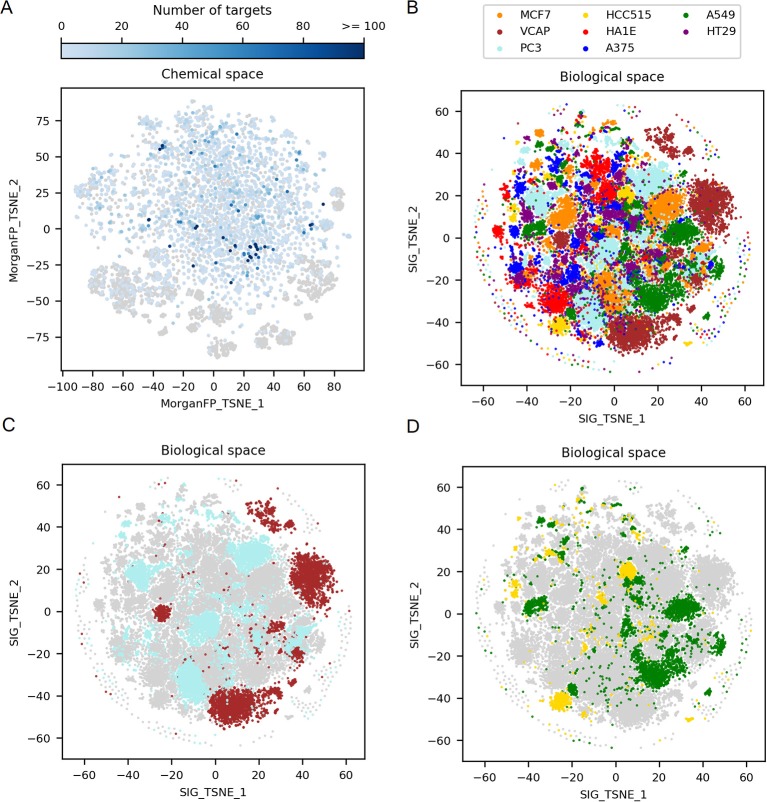
Exploration of the 2D chemical space, along with the corresponding 2D biological space formed by all GES. **(A)** t-SNE on Morgan fingerprints from the 9,035 compounds in working dataset, representing the chemical space. Points corresponding to compounds for which there is no known target are represented by gray points (*n* = 4,163). Points corresponding to compounds for which there is at least one known target are in blue (*n* = 4,872), with darker blue depending on the increasing number of targets. **(B)** t-SNE on all GESs in the working dataset, representing the biological (transcriptomic response) space. Points corresponding to GESs are colored by cell line. **(C)** Biological space highlighting only PC3 and VCAP signatures, 2 cell lines originating from prostate cancer. **(D)** Biological space highlighting only A549 and HCC515 signatures, 2 cell lines originating from lung cancer.

As a first step, to observe the diversity of the 9,035 compounds profiled in the 10 μM/24 h L1000 signature dataset, the corresponding chemical space was visualized. Figure 2A is a 2-dimensional t-SNE representation of the chemical space, illustrating the variability in terms of Morgan fingerprints. The 9,035 compounds form a broad chemical space, with a mean Dice distance between compound pairs of 0.81 (ChEMBL has a mean pairwise Dice distance of 0.82). The center of the chemical space is mostly composed by small molecules having on average a molecular weight lower than 500 Da whereas the outer part is populated by clusters of compounds with higher molecular weights (>500 Da). Overall, we were able to retrieve, in the public domain, at least one activity information for 7,837 compounds, from which 4,872 were active in at least one target. The majority of those compounds were found active in a low number of targets, on average 6 per compound, with a median of 2. Not surprisingly, a set of 23 kinase inhibitors were found to be active in more than 100 targets.

Figure 2B shows a t-SNE plot created using all GESs induced by the 9,035 compounds in the different cell lines to examine the complete biological space. This t-SNE is color coded by the different cell lines used to generate the gene signatures. Each cell line is represented by a set of 4 to 5 main clusters of GESs differing in size and some overlap of the cluster indicates similar GESs derived from different cell lines. In order to better appreciated the differences and communalities in GESs obtained with the selected compounds, t-SNE plots were created highlighting the clusters derived for cell lines originating from the same tumor type namely prostate tumor (VCAP and PC3 in Figure 2C) and lung tumor (A549 and HCC515 in Figure 2D). GESs derived from cell lines coming from the same tissue present very little overlap as can be observed in Figures 2C,D.

These results illustrate the variability in the cellular modifications occurring during carcinogenesis (Hanahan and Weinberg, 2011) and show that each cell line represent a distinct biological space even if the cell lines are derived from the same tissue type. Interestingly, when comparing, for a set of compounds showing GESs in a single cluster in VCAP, GESs of these compounds in PC3 are spread across various clusters from the PC3 biological space (data not shown). This shows that each cell line explores different biological responses to compounds.

**Figure 3:**
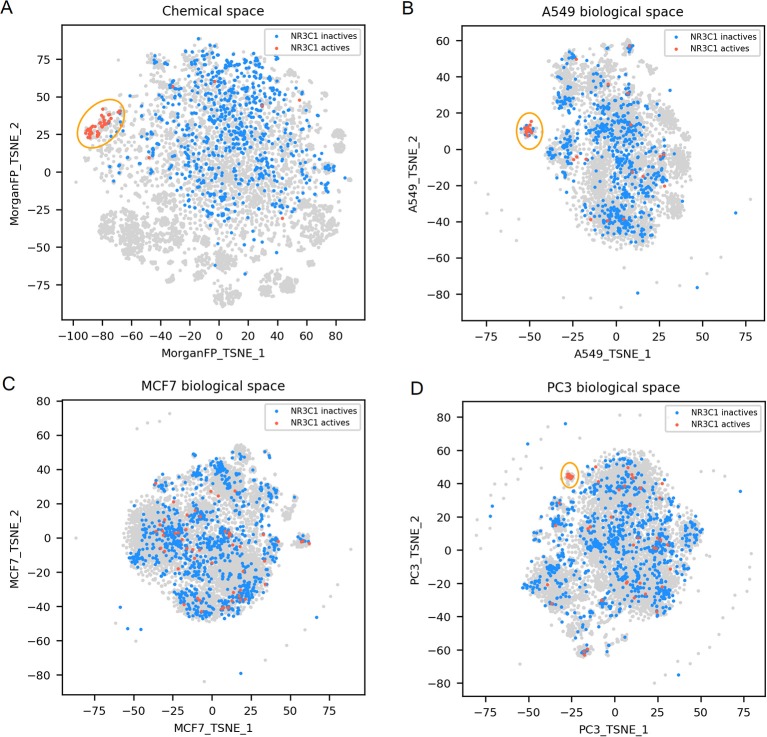
NR3C1 active and inactives compounds in the chemical space and the different biological spaces formed by GES produced in a single cell line. **(A)** Chemical space; **(B)** t-SNE on all A549 signatures (A549 biological space); **(C)** t-SNE on all MCF7 signatures (MCF7 biological space); **(D)** t-SNE on all PC3 signatures (PC3 biological space). Points corresponding to NR3C1 actives are red (*n* = 54), NR3C1 inactives (*n* = 925) are blue, gray points have no available label concerning NR3C1 activity. Orange circles point out clustering of active compounds.

**Figure 4:**
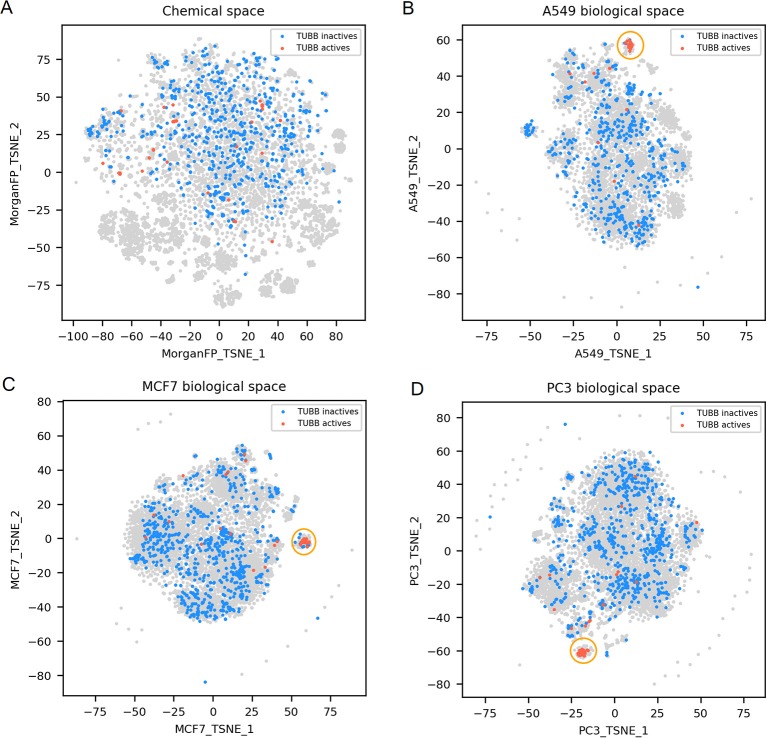
TUBB active and inactives compounds in the chemical space and the different biological spaces formed by GES produced in a single cell line. **(A)** Chemical space; **(B)** A549 biological space; **(C)** MCF7 biological space; **(D)** PC3 biological space. Points corresponding to TUBB actives (*n* = 51) are red, TUBB inactives (*n* = 697) are blue, gray points have no available label concerning TUBB activity. Orange circles point out clustering of active compounds.

**Figure 5:**
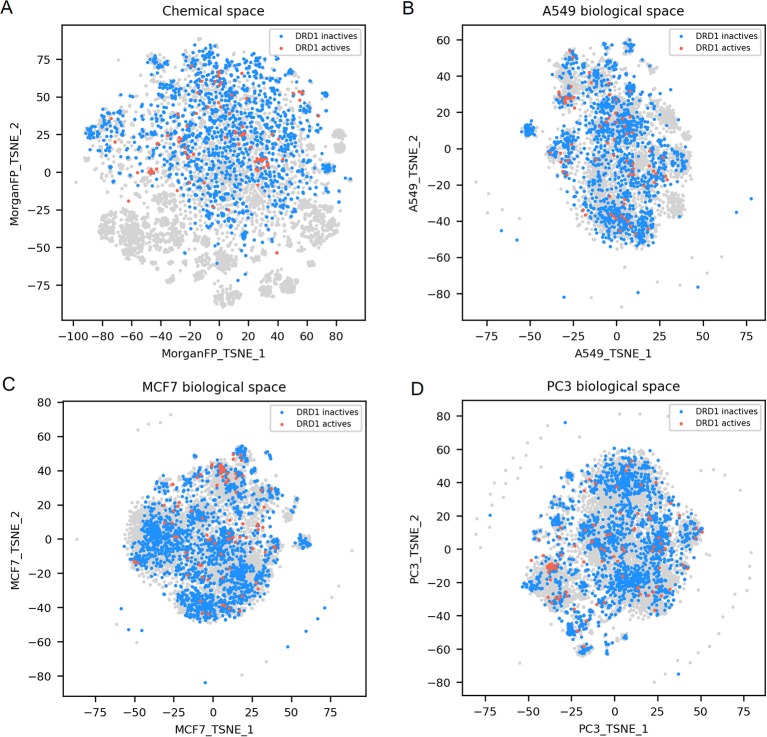
DRD1 active and inactives compounds in the chemical space and the different biological spaces formed by GES produced in a single cell line. **(A)** Chemical space; **(B)** A549 biological space; **(C)** MCF7 biological space; **(D)** PC3 biological space. Points corresponding to DRD1 actives (*n* = 99) are red, DRD1 inactives (*n* = 1843) are blue, gray points have no available label concerning DRD1 activity.

After having described the global variability of GESs in the different cell lines, we explored the chemical and biological spaces corresponding to active and inactive compounds on different targets. Since each compound-induced GES obtained in each cell line was shown to represent a unique biological space, t-SNE plots were computed per cell line in order to further explore the link between the different biological spaces and the corresponding chemical ones. For this, we decided to focus on three cell lines derived from different tissues and among the largest GES dataset generated that is to say A549 (lung cancer), MCF7 (breast cancer) and PC3 (prostate cancer). In addition, we selected 3 representative molecular targets showing different chemical and biological space profiles: compounds active on the glucocorticoid receptor (NR3C1) have similar structures, and similar GESs in some cell lines (Figures 3A–D); tubulin beta I (TUBB) actives have more diverse structures but show similar GESs in each cell line considered in this work (Figures 4A–D); and dopamine receptor D1 (DRD1) actives have diverse structure and GESs in every used cell line (Figures 5A–D).

NR3C1 actives compounds are mostly grouped together in the chemical space, as shown in Figure 3A (*n* = 54; mean Dice distance = 0.67). Most of NR3C1 active GESs are also grouped in a cluster in the A549 biological space (*n* = 38; mean cosine distance = 0.76), visible in Figure 3B, and remaining NR3C1 active GESs are spread across this biological space. Following the similarity principle, we could conjecture that other GESs that are close to this cluster are responses from other NR3C1 actives, especially in the PC3 biological space where the cluster contains compounds known to be active. The same phenomenon can be observed in the biological space of PC3 (Figure 3D), HCC515, HA1E and VCAP (data not shown). Surprisingly, the GES clusters populated by numerous known NR3C1 actives in the biological spaces of A549 and PC3 also contain some known inactive compounds. In the biological spaces of MCF7, A375 and HT29, there is no such clustering, like shown in Figure 3C representing the MCF7 biological space (*n* = 54; mean cosine distance = 0.92). Overall, these results show that compounds that are known to be active on the NR3C1 target can show a similar response in only certain cellular contexts.

TUBB actives compounds are spread in the chemical space (represented in Figure 4A), indicating that they have diverse chemical structures (*n* = 51; mean Dice distance = 0.76). Most importantly, in each cell lines used in this work, GESs induced by TUBB actives compounds were similar (as illustrated in Figures 4B–D), with a rather low mean cosine distance between active compounds ranging between 0.61 and 0.75 depending on the cell line dataset. Moreover, GESs of TUBB actives tend to be similar across all cell lines used in this work (highlighted in Supplementary Figure 1). This conserved pattern in GESs induced by tubulin binding compounds likely illustrate certainly the ubiquitous role of tubulin polymerization of the eukaryotic cytoskeleton (Chaaban and Brouhard, 2017).

Finally, DRD1 actives compounds, that are represented in the chemical space t-SNE, have diverse chemical structures (*n* = 99; mean Dice distance = 0.81), associated with diverse GESs for the 3 cell lines presented (mean cosine distance between 0.88 and 0.92 depending on the cell line), as illustrated in Figures 5A–D. Since GESs of active compounds in any cell lines are not similar, nor their chemical structures, actives cannot be easily discriminated from inactives using these two types of descriptors, as opposed to what was observed with NR3C1 actives that have similar structures, or TUBB actives having similar GESs in every cell line used in this study.

### Model Performances: GES Vs. Morgan Fingerprints

Based on the observed GES similarity of compounds sharing target activity in appropriate cellular contexts, we tested building predictive machine learning models using GESs as descriptors and compare their performances with the ones of the models using Morgan fingerprints.

In order to avoid building models with too unbalanced datasets and to ensure a minimum of active compounds when testing model performances, we first pre-selected targets having at least 50 active compounds in the total dataset (representing between 1 and 63% of active compounds per target). We obtained one dataset per cell line—target combination (restricted to compounds having signatures in the considered cell line, as shown in Figure 1) and carried out a second selection by performing prediction tasks using datasets containing at least 20 active compounds for the considered target (representing between 1 and 69% of active compounds per dataset). For each selected cell line—target dataset, one model using GESs (referred as GES model) was computed. In order to perform a fair comparison per task, one counterpart model using corresponding compound Morgan fingerprints (referred as Morgan FP model) was built using the same set of compounds as the one used in the corresponding GES models. Performances of models were evaluated using the balanced accuracy (BA) metric on a test set, to account for class imbalance in datasets. In total, 990 models were built for a total of 69 different targets. BAs of all built models are presented in Table 3. MCC of all built models are presented in Supplementary Table 1.

**Table 3:** Mean BAs of models (mean per condition).

		**Cell line**	**A375**		**A549**		**HA1E**		**HCC515**		**HT29**		**MCF7**		**PC3**		**VCAP**	
**Target class**	**Target name**	**Target/Descriptor**	**GES**	**Morgan FP**	**GES**	**Morgan FP**	**GES**	**Morgan FP**	**GES**	**Morgan FP**	**GES**	**Morgan FP**	**GES**	**Morgan FP**	**GES**	**Morgan FP**	**GES**	**Morgan FP**
Enzyme	15-Hydroxyprostaglandin dehydrogenase	HPGD	0.5	0.65	0.51	0.7	0.52	0.67	0.5	0.72	0.51	0.68	0.5	0.71	0.52	0.67	0.52	0.64
	Arachidonate 15-lipoxygenase	ALOX15	0.54	0.74	0.57	0.75	0.54	0.67	0.5	0.63	0.5	0.73	0.56	0.74	0.65	0.75	0.53	0.75
	ATP binding cassette B1	ABCB1	0.54	0.62	0.51	0.61	0.5	0.54	0.5	0.5	0.51	0.5	0.56	0.62	0.59	0.63	0.51	0.63
	BRCA1, dna repair associated	BRCA1	0.74	0.69	0.69	0.62	0.67	0.57	0.6	0.52	0.71	0.6	0.78	0.64	0.75	0.64	0.67	0.65
	Cytochrome P450 1A2	CYP1A2	0.54	0.6	0.56	0.6	0.57	0.61	0.59	0.58	0.5	0.59	0.59	0.63	0.6	0.63	0.56	0.61
	Cytochrome P450 2C19	CYP2C19	0.52	0.57	0.55	0.59	0.5	0.55	0.52	0.55	0.51	0.57	0.56	0.58	0.56	0.59	0.53	0.58
	Cytochrome P450 2C9	CYP2C9	0.55	0.57	0.52	0.61	0.52	0.6	0.51	0.6	0.53	0.58	0.55	0.58	0.6	0.58	0.54	0.6
	Cytochrome P450 3A4	CYP3A4	0.51	0.55	0.52	0.59	0.52	0.58	0.49	0.6	0.51	0.54	0.52	0.6	0.54	0.6	0.53	0.57
	DNA polymerase beta	POLB	0.5	0.66	0.5	0.69	0.51	0.82	–	–	0.5	0.69	0.5	0.69	0.53	0.73	0.5	0.69
	DNA polymerase eta	POLH	0.55	0.71	0.51	0.74	0.66	0.82	–	–	0.5	0.76	0.51	0.73	0.55	0.72	0.52	0.69
	DNA polymerase iota	POLI	0.5	0.7	0.5	0.71	0.54	0.71	0.5	0.6	0.51	0.65	0.51	0.72	0.51	0.73	0.5	0.68
	DNA polymerase kappa	POLK	0.58	0.77	0.51	0.78	0.56	0.83	0.52	0.79	0.5	0.76	0.54	0.81	0.56	0.83	0.5	0.83
	Flap structure-specific endonuclease 1	FEN1	0.5	0.72	0.5	0.68	0.5	0.77	–	–	0.5	0.74	0.5	0.77	0.51	0.75	0.5	0.7
	Glutaminase	GLS	0.5	0.64	0.51	0.64	0.53	0.58	–	–	–	–	0.51	0.74	0.51	0.69	0.51	0.7
	Growth factor, augmenter of liver regeneration	GFER	0.51	0.72	0.5	0.72	0.59	0.74	0.5	0.71	0.5	0.72	0.52	0.72	0.56	0.71	0.5	0.7
	Hydroxysteroid 17-beta dehydrogenase 10	HSD17B10	0.51	0.57	0.54	0.62	0.55	0.63	0.51	0.63	0.49	0.58	0.54	0.65	0.53	0.64	0.5	0.62
	Janus kinase 2	JAK2	0.71	0.57	0.73	0.63	0.69	0.56	0.63	0.56	0.68	0.56	0.8	0.63	0.73	0.59	0.65	0.58
	MDM2 proto-oncogene	MDM2	0.77	0.69	0.71	0.61	0.7	0.62	0.76	0.53	0.67	0.59	0.83	0.65	0.81	0.64	0.76	0.66
	Phosphatidylinositol-5-phosphate 4-kinase type 2 alpha	PIP4K2A	0.51	0.69	0.54	0.75	0.53	0.66	–	–	0.5	0.58	0.53	0.76	0.5	0.76	0.52	0.74
	Phospholipase A2 group VII	PLA2G7	0.5	0.71	0.51	0.65	0.57	0.72	–	–	0.5	0.69	0.54	0.69	0.57	0.71	0.51	0.69
	Polo like kinase 1	PLK1	0.5	0.62	0.52	0.64	0.54	0.52	0.49	0.56	0.5	0.52	0.52	0.68	0.52	0.67	0.49	0.68
	Serine/threonine kinase 33	STK33	0.78	0.58	0.68	0.62	0.78	0.59	0.74	0.6	0.7	0.56	0.72	0.61	0.71	0.64	0.66	0.65
	Ubiquitin specific peptidase 1	USP1	0.5	0.54	0.51	0.58	0.51	0.53	0.51	0.53	0.5	0.55	0.52	0.57	0.57	0.59	0.5	0.57
	YES proto-oncogene 1, Src family tyrosine kinase	YES1	0.7	0.72	0.67	0.7	0.71	0.72	0.63	0.69	0.66	0.71	0.66	0.75	0.7	0.72	0.54	0.68
Epigenetic regulator	Bromodomain adjacent to zinc finger domain 2B	BAZ2B	0.6	0.66	0.54	0.68	0.63	0.69	0.52	0.6	0.5	0.65	0.55	0.67	0.6	0.66	0.53	0.68
	Chromobox 1	CBX1	0.56	0.57	0.55	0.62	0.6	0.6	0.57	0.61	0.54	0.55	0.61	0.6	0.57	0.62	0.55	0.61
	Lysine demethylase 4A	KDM4A	0.64	0.62	0.56	0.67	0.65	0.7	0.57	0.63	0.6	0.65	0.58	0.68	0.6	0.65	0.53	0.63
	Lysine demethylase 4E	KDM4E	0.59	0.76	0.52	0.75	0.61	0.75	0.53	0.75	0.5	0.72	0.53	0.73	0.57	0.74	0.52	0.72
	M-phase phosphoprotein 8	MPHOSPH8	0.52	0.53	0.52	0.64	0.54	0.64	0.51	0.65	0.5	0.54	0.63	0.63	0.54	0.64	0.51	0.65
	Protein arginine methyltransferase 1	PRMT1	0.5	0.65	0.51	0.71	0.5	0.54	–	–	–	–	0.52	0.7	0.51	0.71	0.51	0.72
	Sirtuin 5	SIRT5	–	–	0.51	0.65	–	–	–	–	–	–	0.51	0.62	0.5	0.61	0.5	0.6
	Survival of motor neuron 2, centromeric	SMN2	–	–	0.5	0.62	0.51	0.56	–	–	–	–	0.52	0.63	0.52	0.6	0.5	0.62
Ion channel	Potassium voltage-gated channel H2	KCNH2	0.72	0.82	0.64	0.8	0.66	0.79	0.65	0.76	0.74	0.8	0.65	0.84	0.65	0.8	0.67	0.8
Membrane receptor	5-hydroxytryptamine receptor 1A	HTR1A	0.5	0.72	0.51	0.75	0.54	0.75	0.53	0.75	0.51	0.7	0.55	0.77	0.52	0.76	0.55	0.79
	Cholinergic receptor muscarinic 1	CHRM1	0.6	0.69	0.64	0.69	0.66	0.71	0.7	0.69	0.67	0.62	0.59	0.71	0.6	0.73	0.65	0.72
	Cholinergic receptor muscarinic 4	CHRM4	0.63	0.71	0.67	0.75	0.64	0.72	0.7	0.68	0.63	0.66	0.66	0.7	0.62	0.73	0.62	0.72
	Cholinergic receptor muscarinic 5	CHRM5	0.6	0.69	0.62	0.76	0.64	0.72	0.69	0.72	0.63	0.68	0.64	0.73	0.59	0.71	0.61	0.68
	Dopamine receptor D1	DRD1	0.64	0.68	0.6	0.73	0.63	0.7	0.58	0.72	0.62	0.69	0.62	0.74	0.61	0.72	0.59	0.74
	Dopamine receptor D2	DRD2	0.61	0.74	0.61	0.79	0.61	0.79	0.6	0.79	0.61	0.73	0.65	0.79	0.62	0.79	0.63	0.8
	Dopamine receptor D3	DRD3	0.6	0.66	0.58	0.72	0.56	0.71	0.58	0.71	0.58	0.66	0.63	0.72	0.57	0.72	0.58	0.73
	Neuropeptide S receptor 1	NPSR1	–	–	0.59	0.64	0.5	0.55	–	–	–	–	0.63	0.66	0.57	0.66	0.58	0.63
	Opioid receptor kappa 1	OPRK1	0.5	0.61	0.52	0.65	0.5	0.61	–	–	0.54	0.63	0.57	0.65	0.53	0.64	0.55	0.68
	Thyroid stimulating hormone receptor	TSHR	0.51	0.64	0.5	0.56	0.56	0.52	0.57	0.5	–	–	0.56	0.61	0.56	0.6	0.55	0.61
	TNF receptor superfamily member 10b	TNFRSF10B	–	–	0.69	0.61	0.56	0.52	–	–	0.7	0.56	0.71	0.61	0.78	0.58	0.65	0.56
Other cytosolic protein	Heat shock protein 90 alpha A1	HSP90AA1	0.53	0.65	0.5	0.67	0.59	0.73	–	–	0.53	0.67	0.55	0.67	0.59	0.65	0.51	0.64
	Heat shock protein family B1	HSPB1	0.58	0.58	0.54	0.53	0.63	0.58	0.54	0.51	0.5	0.55	0.66	0.59	0.66	0.61	0.55	0.57
Secreted protein	Interleukin 1 beta	IL1B	0.62	0.55	0.65	0.6	0.65	0.55	0.65	0.55	0.66	0.57	0.68	0.63	0.69	0.62	0.63	0.62
Structural protein	Tubulin beta class I	TUBB	–	–	0.81	0.8	0.82	0.78	–	–	–	–	0.88	0.82	0.84	0.8	0.82	0.8
Transcription factor	Androgen receptor	AR	0.51	0.63	0.58	0.75	0.55	0.71	0.53	0.75	0.51	0.62	0.61	0.77	0.55	0.74	0.67	0.76
	Jun proto-oncogene, AP-1 transcription factor subunit	JUN	0.6	0.69	0.54	0.63	0.58	0.65	0.56	0.61	0.59	0.67	0.6	0.65	0.57	0.67	0.6	0.63
	Melanogenesis associated transcription factor	MITF	0.81	0.64	0.7	0.61	0.73	0.57	0.73	0.56	0.68	0.57	0.82	0.65	0.79	0.68	0.69	0.61
	Nuclear factor kappa B1	NFKB1	0.51	0.51	0.5	0.66	0.51	0.5	0.5	0.51	0.55	0.5	0.5	0.63	0.51	0.64	0.51	0.63
	Nuclear receptor 3C1	NR3C1	–	–	0.77	0.96	0.67	0.94	0.76	0.98	–	–	0.6	0.93	0.73	0.95	0.69	0.95
	Nuclear receptor 5A1	NR5A1	0.55	0.53	0.65	0.56	0.64	0.57	–	–	–	–	0.72	0.62	0.73	0.62	0.65	0.6
	Tumor protein p53	TP53	0.72	0.57	0.62	0.55	0.65	0.55	0.7	0.57	0.62	0.58	0.71	0.58	0.7	0.57	0.6	0.56
	Vitamin D receptor	VDR	0.5	0.57	0.5	0.6	0.52	0.6	0.51	0.54	0.53	0.53	0.58	0.62	0.55	0.59	0.53	0.59
Transporter	Abhydrolase domain containing 5	ABHD5	0.51	0.57	0.51	0.66	–	–	–	–	–	–	0.55	0.68	0.54	0.68	0.53	0.69
	Solute carrier family 6 member 3	SLC6A3	0.64	0.65	0.62	0.66	0.65	0.65	0.67	0.62	0.61	0.65	0.66	0.66	0.66	0.67	0.64	0.68
Unclassified protein	Ataxin 2	ATXN2	0.78	0.5	0.7	0.62	0.74	0.52	0.7	0.53	0.69	0.58	0.72	0.62	0.72	0.61	0.7	0.61
	ATPase family, AAA domain containing 5	ATAD5	0.58	0.56	0.52	0.67	0.59	0.62	0.55	0.62	0.52	0.6	0.6	0.65	0.64	0.65	0.52	0.68
	Endothelial PAS domain protein 1	EPAS1	–	–	0.63	0.62	–	–	–	–	–	–	0.73	0.68	0.76	0.65	0.69	0.67
	Geminin, DNA replication inhibitor	GMNN	0.7	0.59	0.71	0.58	0.69	0.55	0.65	0.59	0.67	0.55	0.76	0.62	0.75	0.59	0.68	0.6
	MLLT3, super elongation complex subunit	MLLT3	–	–	0.5	0.54	–	–	–	–	–	–	0.55	0.55	0.51	0.63	0.51	0.67
	MYC proto-oncogene, bHLH transcription factor	MYC	–	–	0.73	0.65	–	–	–	–	–	–	0.69	0.65	0.63	0.66	0.77	0.64
	Nuclear factor, erythroid 2 like 2	NFE2L2	0.57	0.51	0.57	0.6	0.55	0.58	0.57	0.59	0.58	0.53	0.56	0.61	0.61	0.6	0.59	0.6
	Nucleotide binding oligomerization domain containing 1	NOD1	–	–	0.58	0.66	–	–	–	–	0.6	0.58	0.66	0.69	0.65	0.68	0.56	0.7
	Nucleotide binding oligomerization domain containing 2	NOD2	0.5	0.53	0.53	0.61	0.56	0.54	–	–	0.52	0.53	0.58	0.68	0.61	0.68	0.56	0.64
	RAD52 homolog, DNA repair protein	RAD52	–	–	0.54	0.57	–	–	–	–	–	–	0.52	0.61	0.53	0.63	0.53	0.69
	TAR DNA binding protein	TARDBP	–	–	0.5	0.56	0.51	0.54	–	–	–	–	0.54	0.55	0.54	0.54	0.5	0.57

*Target in lines, and cell line and used descriptor in columns. GES, model using gene expression signature. Morgan FP, model using chemical fingerprints from counterpart GES model dataset. Cells containing “-” corresponds to models that were not computed in cause of a too low number of actives (<20) in the dataset to perform appropriate classification. Model presenting BA between 0.7 and 0.8 are highlighted in orange whereas those with BA >0.8 are highlighted in red*.

Overall, GES model performances appeared to be variable depending on the predicted target and on the cell line that was used to generate the GESs, with a BA ranging from 0.49 to 0.88. Counterpart models trained with Morgan fingerprints also had variable performances, with a BA ranging from 0.50 to 0.98. On average, Morgan FP models (mean BA = 0.65) yielded better performances for the target activity prediction than their counterpart GES models (mean BA = 0.58). On the 495 cell line—target combinations, BA of GES models was higher than BA of counterpart Morgan FP models for 124 combinations (25%).

On the 990 models, 208 models reached a BA higher than 0.7 (21%) for 40 targets (59 GES models for 18 targets; 138 Morgan FP models for 28 targets), and 33 models reached a BA higher than 0.8 (3%) for 10 targets (10 GES models for 4 targets; 21 Morgan FP models for 7 targets). For all 138 Morgan FP models reaching BA higher than 0.7, BA was superior to counterpart GES models, and for the 59 GES models reaching BA higher than 0.7, only 6 had counterpart Morgan FP model with higher BA.

For NR3C1 activity prediction, Morgan FP models yielded a BA between 0.93 and 0.98 depending on the cell line dataset. It is not surprising considering that that NR3C1 actives have similar structure as shown in Figure 3A. On the GES models, a BA of 0.77 was reached using the A549 signature dataset, correlating to similar GESs that were observed in the A549 biological space (Figure 3B), whereas a BA of 0.6 was obtained using the MCF7 signature dataset (no GES cluster in MCF7 biological space, shown in Figure 3C). A549 and MCF7 signature model performances cannot be fairly compared because they were built using different sets of compounds. In fact, performances of different GES models cannot be compared across cell lines nor across targets, performances can only be compared to observed similarity between active compounds in either chemical and biological space plots for a given target.

For TUBB activity prediction, GES models yielded BA between 0.81 and 0.88 depending on the cell line dataset, which was among the 10 best GES models. Interestingly, their counterpart Morgan FP models were not significantly underperforming (BA ranging from 0.78 to 0.82). Even though the TUBB active structures are diverse, the models still managed to identify structural fragments that could produce such predictive performance.

For DRD1 activity prediction, Morgan FP models yielded BA between 0.68 and 0.74 depending on the cell line dataset, and were always better than their counterpart GES model, with a BA ranging from 0.58 to 0.64.

Overall, we conclude that it was possible to build GES models with acceptable performances, performing similarly or better than their counterpart Morgan FP models in 25% of the target prediction tasks. Moreover, we see an important advantage in the GES models: they are theoretically performing independently of the chemical space considered, allowing target identification of new compounds even if their corresponding structural diversity is not represented in the training set.

### Rationalizing Model Performances Using Distance Plots

To further describe and understand the reasons for the differences in performances between GES model and Morgan FP model, for every dataset used in each cell line—target combinations, Morgan fingerprints Dice distance was plotted against GES cosine distance between each pair of active compounds in the given dataset.

Generated distance plots were split in 4 quadrants separated by a 0.5 threshold for Dice distance (dotted vertical line) and a 0.5 threshold for cosine distance (dotted horizontal line). Data points in top right (Quadrant I) represent pairs of active compounds showing diverse structures and different GESs in the considered cell line and contains most of compound pairs (average of 95.1%). Data points in top left (Quadrant II) represent pairs of active compounds showing similar structures while showing diverse GESs in the considered cell line (average of 1.9%). Data points in bottom left (Quadrant III) represents pairs of active compounds showing similar structures and similar GESs in the considered cell line and contains least compound pairs (average of 0.5%). Data points in bottom right (Quadrant IV) represents pairs of active compounds showing similar GESs while having different structures (average of 2.5%). Intuitively, we think that sample similarity within the same class (here: actives) is a good indicator to know if a machine learning model will be able to properly predict samples from this class.

Overall, the mean percentage of compound pairs (active and inactives) were 99.3, 0.3, 0.01 and 0.4% for quadrants I, II, III and IV respectively. Based on this dataset, compounds active toward a molecular target have on average more similar structures and GESs than the totality of the compounds.

We expected to reach good Morgan FP model prediction for combinations having a high proportion of points in quadrants II and III (similar structures), and good GES model prediction for combinations having a high proportion of points in quadrants III and IV (similar GESs). We evaluated the use of distance plots on the three targets and three cell lines used in previous space plots (Figures 3, 5). Similar work was performed using not only active compounds, but all compounds having at least one annotation for each of the three previously described targets, shown in Supplementary Figure 2.

**Figure 6:**
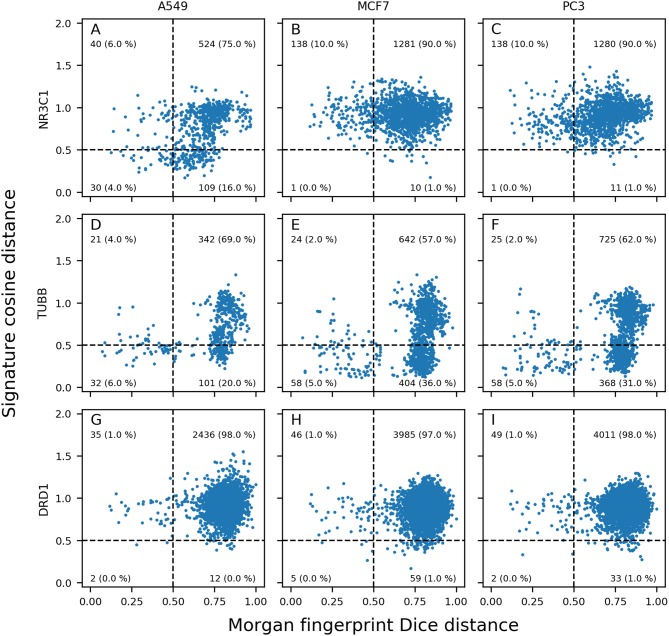
Morgan fingerprints Dice distance vs. GES cosine distance (distance plots). Different panels show information for pairs of NR3C1 **(A–C)**, TUBB **(D–F)**, and DRD1 **(G–I)**; active compounds in A549 **(A,D,G)**, MCF7 **(B,E,H)**, and PC3 **(C,F,I)** cell lines.

For NR3C1 distance plots, there are approximately 10% of compound pairs in quadrants II and III of the 3 plots (Figures 6A–C), coherent with good Morgan FP model performances. However, depending on which cell line the GESs were generated from, there were different proportions of compound pairs in quadrants III and IV: there are 20% of pairs for A549, and only 1% of pairs in MCF7. This is in agreement with what was observed in model performances: performance of GES models using the A549 dataset (BA = 0.77) was much better than performances using MCF7 dataset (BA = 0.60). Surprisingly, prediction using GESs from the PC3 dataset showed good performances (BA = 0.73), even though the proportion of active compound pairs in quadrants III and IV was around 1% (similar to the proportion observed for the MCF7 dataset that showed worse performances). This suggests that the GES model built with PC3 was able to capture a subset of genes to discriminate active compounds from inactives, even with active compounds showing different GESs.

For TUBB distance plots (Figures 6D–F), between 7 and 10% of compound pairs was observed in quadrants II and III, matching the good Morgan FP model performances with the 3 cell line datasets (BA ranging from 0.80 to 0.82). Most importantly, there are between 26 and 40% of compound pairs in quadrants III and IV, echoing the better performances of the GES models in these cell lines (BA ranging from 0.81 to 0.88).

For DRD1 distance plots (Figures 6G–I), 98% of compound pairs are in quadrant I, leaving low number of active compound pairs in the other quadrants (with similar Morgan fingerprints and/or GESs). This is coherent with the average performances of GES (BA ranging from 0.68 to 0.74) and Morgan FP models (BA ranging from 0.58 to 0.64) built for this target.

Among the 50 best GES models, the mean percentage of active pairs in quadrants III and IV was 5.2% (vs. 2.3% in quadrants II and III). For the 50 best Morgan FP models, the mean percentage of active pairs in quadrants II and III was 4.0% (vs. 2.2% in quadrants III and IV). This suggests a positive relationship between sample similarity between active compounds using a given set of descriptors for active compounds and performances of models using these descriptors.

In the current work, GESs were shown to be effective descriptors to predict compound activity toward molecular targets. In 25% of target prediction tasks, GES models outperformed their counterpart Morgan FP models, especially when using GES produced in a cell line exhibiting similar GESs between compounds active toward the target of interest. Such GES models performs independently of the structural diversity of compounds that were used to produce GESs, offering a great opportunity to escape the classical chemical space limitations associated with QSAR models. In addition, t-SNE plots, along with 2D distance plots, can give insights to assess the predictive power of GESs and Morgan fingerprints for target prediction, based on a limited dataset depending on biological (GES and bioactivity assay) data availability.

## Discussion

Our results show that compound-induced transcriptomic responses derived from cell lines have the potential to support target prediction of unknown compounds with large structural diversity. Interestingly, we observed that compound induced biological responses are mostly cell line specific even when cell lines are derived from the same tissue. Nevertheless, machine learning models using GESs were shown to perform well as long as the appropriate cell line was used. Exploring biological spaces can help to overcome the limitations derived from a restricted chemical space when using traditional QSAR. To improve the predictivity of GES models, we have identified several limitations, and discuss possible improvements.

### Data Acquisition

First limitations come from gene expression data preprocessing. Gene expression values were obtained through multiple preprocessing steps from the initial generated raw data. For instance, there is a first peak deconvolution step to determine the gene expression levels, that as well as the plate-normalized z-scoring to obtain the normalized (“Level 5”) can still be improved as already stated by Li et al. (2017). Using GESs obtained with different preprocessing methods could potentially give more accurate normalized values leading to increased performances in machine learning models.

Secondly, the CMAP L1000 technology relies on the measurement of 978 landmark genes, representing about 5% of the human transcriptome (Pertea, 2012). The gene values of the remaining transcriptome can be inferred through different computational methods (Subramanian et al. (2017) method reached good prediction for 81% of inferred genes), that are still under improvement (Blasco et al., 2019). We decided to only use the 978 landmarks as input data for the machine models generated, to reflect real measured gene expression. Doing so, we might have missed some valuable information captured by a change of expression of the non-measured genes. Therefore, it would be interesting to explore the potential added value of expanding the number of descriptors by adding the inferred gene information to the target prediction models.

### Data Restrictions

Another limitation is also coming with the activity dataset that was used. Since compound activity is a selective interaction, there is for each target a low number of active compounds compared to the number of inactive compounds. As a consequence, the training sets used for model building were highly unbalanced favorizing the prediction of the category inactive. Moreover, not every compound was tested for activity in all targets, leading to a sparse dataset (5% of total compound target interactions are known).

On top of this activity data limitation, not all available compounds were profiled in all the 8 cell lines used in this work. There were only about 600 compounds profiled in all the cell lines, which is too limited to build predictive models, with regard to available activity data. Consequently, one dataset per cell line was created, formed by compounds profiled in this cell line and resulting GESs. For each target prediction, the cell line datasets were restricted to compounds having a known label for the target of interest. Since each task used a different dataset, performances of models across cell lines or targets the comparison across GES models was not possible. The difference in dataset sizes is explaining at least partly the variation of performances of GES models across targets, ranging from models close to a random predictor (BA = 0.50) to good GES models (BA = 0.88), as well as the variation of performances of counterpart Morgan FP models (BA ranging from 0.50 to 0.98).

### Biological Response Constraints

Biologically, variation of GES model performances can also be caused by the difference in the pathway representation in the cell lines and consequently to compound induced signatures. Compounds active on a given target might show GESs with different degree of similarity or no similarity among the considered cell lines. as illustrated by the cases of NR3C1, TUBB and DRD1. Gene expression responses depend on the cellular context as shown in this work and elsewhere (Chen et al., 2013; Yu et al., 2019). Thus, the biological system in which the GESs are generated is of utmost importance for target prediction.

Due to practical aspects (scalability, low price, etc.), biological systems such as *in vitro* immortalized cell cultures (like cancer cell lines used in this work) are widely used, but they come with some disadvantages: they show limited physiological representativity and have been shown to drift along passages (Hughes et al., 2007). Even within the same cell line, it was shown that strains show different responses to the same compounds, indicating a reduced reproducibility between generated GESs (Ben-David et al., 2018). Ideally, the GESs should be derived from biological systems mimicking as much as possible the biological responses observed in the corresponding target organ.

The advantage of transcriptomic evaluations over single endpoint assays is that in theory they have the potential to capture integrative responses from compound treatments, ranging from on target activity at high potency to off-target activities at lower potencies, depending on the tested concentrations. GESs responses are also known to be variable depending on time exposition (Aguayo-Orozco et al., 2018). That is the reason why we selected data sets originating from the same study design. GESs measured at a concentration of 10 μM after 24 h of treatment of the cell lines were extracted, as this is the most represented experimental condition (De Wolf et al., 2016; Lv et al., 2017).

### GES Models Versus Morgan FP Models

We showed that using GES datasets produced by the Broad Institute with the CMAP L1000 technique (Subramanian et al., 2017), random forest models outperformed counterpart Morgan FP models for target prediction in 25% of the cases. Evidently, the outcome of this comparison is depending on the available data for the different targets to build the models as illustrated by the wide range of differences of BA between the two types of predictive models. Practically, both QSAR and transcriptomic descriptors represent good opportunities for target prediction, but each come with advantages and constraints that needs to be considered when building predictive models.

QSAR models for target prediction are widely used because of the wide dataset available, with existing databases like PubChem or ChEMBL. Most QSAR descriptors are discrete unambiguous values extracted from the chemical formula of compounds, thus easily computed. In the context of hit discovery, a major drawback of QSAR models is that they show significant error rate when trying to predict activity for compounds that are too structurally different from the training set (Cherkasov et al., 2014). Using a new set of descriptors, like compound bioactivity such as GESs extracted from *in vitro* experiments, can help in target prediction while escaping from the classical chemical space limitation observed in QSAR approaches.

On the other hand, GESs represents a number of changes on a certain number of genes (the 978 landmarks), capturing the effect of compounds. These data could be used to make inference about biology (i.e., finding targets or biomarkers). Each cell line shows a unique biological space that can be explored. However, these biological experiment data are prone to technical and biological variability like discussed earlier. Gene expression can be measured in different dose and time conditions, adding dimensions to explore in order to find the conditions reaching best performances in GES models. Finally, the gene expression measurements are more and more cost effective, making the use of such data at a large scale possible.

When exploring a new chemical class in hit discovery, evaluating chemical-induced biological responses in appropriate cell-lines using transcriptomic profiling can support chemical prioritization. This biologically-based approach present the advantage in a given biological space of being in principle chemical space independent as opposed to QSAR modeling that is constrained by the chemical space of the training set. Furthermore, during lead optimization, biological spaces inform about the direct activity of candidates, which can help fine-tuning their desired activity profile, by optimizing the on-target activity. It has been recently shown that this type of data can be used for *de novo* chemical design fulfilling a specific GES (Méndez-Lucio et al., 2020). In a chemical safety approach, it can be used to detect compound interaction with off-targets. However, a difference between these 2 applications would be the conditions in which the GESs are generated: on-target effects are observable at low concentrations (Kd often in the nanomolar range), while off-target effect are known to typically appear at higher concentration as illustrated by Li et al. (2019).

In conclusion, in this work, we evaluated the use of a large public dataset of compound-induced transcriptomic data, to predict compound activity on 69 molecular targets. We compared machine learning models built with transcriptomics data with counterpart models built using Morgan fingerprints. Active compounds on a given target could exhibit similar signatures in one or multiple cell lines, independent from the chemical structure similarity between these active compounds. For 25% of the tasks, random forest models using transcriptomics signatures performed similarly or better than counterpart models built with Morgan fingerprints, occurring mostly using signatures produced in cell lines that showed similar signatures for active compounds on a given target. Compound-induced transcriptomic data could offer a great opportunity for target prediction based on cell response similarity and allows to circumvent the applicability domain limitation of QSAR models.

## Data Availability Statement

Publicly available datasets were analyzed in this study. This data can be found in the Gene Expression Omnibus https://www.ncbi.nlm.nih.gov/gds (GEO IDs GSE92742 and GSE70138).

## Author Contributions

BB, OM-L, and DR contributed to the conception of the work. BB performed the workflow with the help of OM-L. BB wrote the first draft of the manuscript. JW, OM-L, and DR provided guidance and helped with the manuscript preparation and contributed to manuscript revision, read and approved the submitted version. DR and OM-L wrote sections of the manuscript.

## Conflict of Interest

JW is employee of Bayer AG. OM-L, BB, and DR work directly or indirectly for Bayer SAS.
