# Supplementary material for: Exploring the Use of Compound-Induced Transcriptomic Data Generated From Cell Lines to Predict Compound Activity Toward Molecular Targets
Source: Front Chem. 2020 Apr 23;8:296. doi: 10.3389/fchem.2020.00296 (PMC7191531; doi:10.3389/fchem.2020.00296)
Supplement: Supplementary file 1 [file Table_1.DOCX]

Supplementary Material

## Supplementary Figures


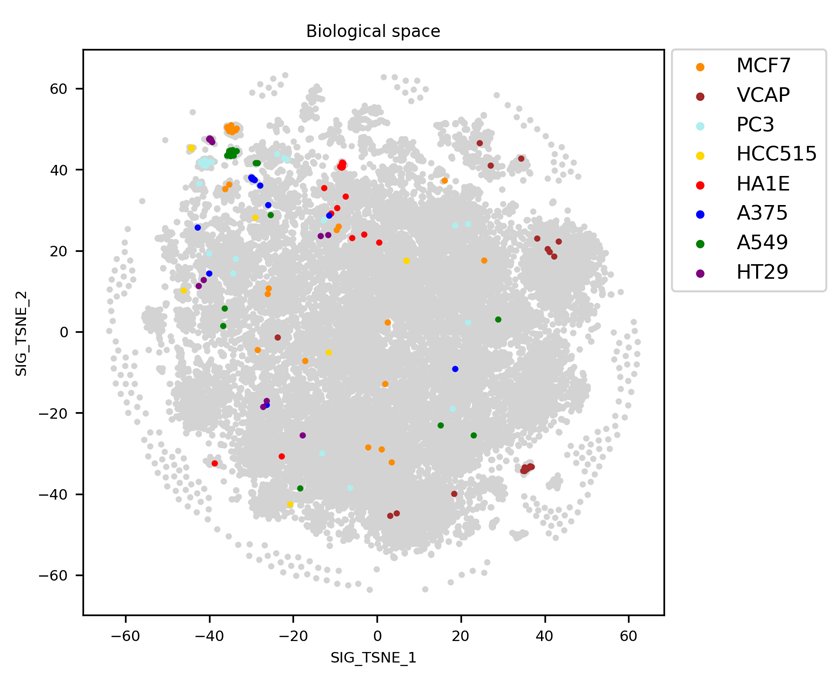


**Supplementary Figure 1:** t-SNE on all GESs in the working dataset, representing the biological (transcriptomic response) space. Points corresponding to GESs of known TUBB actives are colored by cell line, points corresponding to other GESs are colored in grey.


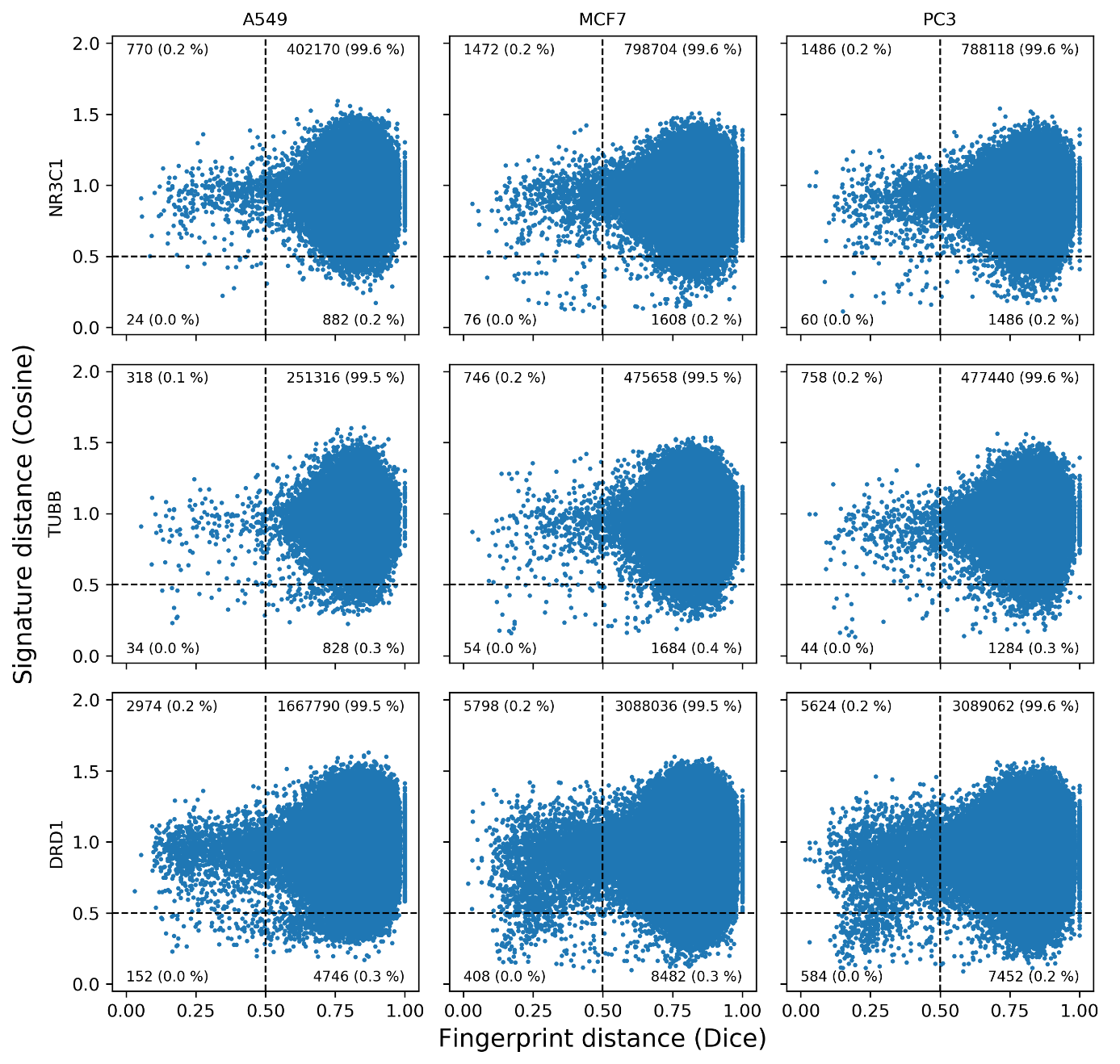


**Supplementary Figure 2**: Morgan fingerprints Dice distance versus GES cosine distance (distance plots) for pairs of compounds having at least on annotation in NR3C1 TUBB and DRD1, in A549, MCF7 and PC3 cell lines

## Supplementary tables

Supplementary Table 1: Mean MCCs of models (mean per condition). Target in lines, and cell line and used descriptor in columns. GES: model using gene expression signature. Morgan FP: model using chemical fingerprints from counterpart GES model dataset. Cells containing “-” corresponds to models that were not computed in cause of a too low number of actives (< 20) in the dataset to perform appropriate classification.

|  |  | Cell line | A375 | | A549 | | HA1E | | HCC515 | | HT29 | | MCF7 | | PC3 | | VCAP | |
| --- | --- | --- | --- | --- | --- | --- | --- | --- | --- | --- | --- | --- | --- | --- | --- | --- | --- | --- |
| Target class | Target name | Target / Descriptor | GES | Morgan FP | GES | Morgan FP | GES | Morgan FP | GES | Morgan FP | GES | Morgan FP | GES | Morgan FP | GES | Morgan FP | GES | Morgan FP |
| Enzyme | 15-hydroxyprostaglandin dehydrogenase | HPGD | 0 | 0.29 | 0.01 | 0.32 | 0.07 | 0.29 | -0.02 | 0.39 | 0.05 | 0.3 | 0.01 | 0.31 | 0.05 | 0.26 | 0.05 | 0.23 |
|  | arachidonate 15-lipoxygenase | ALOX15 | 0.16 | 0.51 | 0.2 | 0.57 | 0.17 | 0.37 | -0.01 | 0.26 | 0.03 | 0.52 | 0.21 | 0.54 | 0.4 | 0.54 | 0.07 | 0.56 |
|  | ATP binding cassette B1 | ABCB1 | 0.13 | 0.29 | 0.05 | 0.26 | 0.01 | 0.09 | 0 | -0.01 | 0.01 | 0 | 0.13 | 0.33 | 0.22 | 0.32 | 0.02 | 0.38 |
|  | BRCA1, DNA repair associated | BRCA1 | 0.36 | 0.34 | 0.3 | 0.17 | 0.3 | 0.11 | 0.18 | 0.04 | 0.33 | 0.18 | 0.37 | 0.22 | 0.39 | 0.24 | 0.36 | 0.29 |
|  | cytochrome P450 1A2 | CYP1A2 | 0.08 | 0.2 | 0.12 | 0.2 | 0.12 | 0.21 | 0.19 | 0.15 | -0.01 | 0.18 | 0.17 | 0.27 | 0.19 | 0.25 | 0.13 | 0.2 |
|  | cytochrome P450 2C19 | CYP2C19 | 0.04 | 0.14 | 0.1 | 0.17 | -0.01 | 0.1 | 0.04 | 0.09 | 0.02 | 0.14 | 0.11 | 0.17 | 0.13 | 0.18 | 0.06 | 0.17 |
|  | cytochrome P450 2C9 | CYP2C9 | 0.11 | 0.15 | 0.04 | 0.21 | 0.05 | 0.2 | 0.01 | 0.22 | 0.07 | 0.17 | 0.11 | 0.17 | 0.21 | 0.16 | 0.09 | 0.2 |
|  | cytochrome P450 3A4 | CYP3A4 | 0.02 | 0.1 | 0.04 | 0.14 | 0.06 | 0.15 | -0.03 | 0.2 | 0.02 | 0.07 | 0.05 | 0.16 | 0.08 | 0.16 | 0.07 | 0.12 |
|  | DNA polymerase beta | POLB | 0 | 0.35 | -0.02 | 0.32 | 0.05 | 0.47 |  |  | -0.01 | 0.38 | 0.01 | 0.34 | 0.07 | 0.36 | 0 | 0.3 |
|  | DNA polymerase eta | POLH | 0.14 | 0.38 | 0.05 | 0.39 | 0.44 | 0.52 |  |  | -0.01 | 0.44 | 0.03 | 0.33 | 0.11 | 0.35 | 0.08 | 0.32 |
|  | DNA polymerase iota | POLI | 0 | 0.39 | -0.01 | 0.33 | 0.17 | 0.37 | -0.01 | 0.26 | 0.02 | 0.28 | 0.03 | 0.35 | 0.03 | 0.37 | 0.02 | 0.3 |
|  | DNA polymerase kappa | POLK | 0.14 | 0.41 | 0.04 | 0.34 | 0.21 | 0.43 | 0.05 | 0.39 | 0 | 0.38 | 0.1 | 0.43 | 0.12 | 0.41 | 0.01 | 0.39 |
|  | flap structure-specific endonuclease 1 | FEN1 | 0 | 0.33 | 0 | 0.28 | -0.01 | 0.35 |  |  | -0.01 | 0.37 | 0 | 0.37 | 0.03 | 0.32 | 0 | 0.3 |
|  | glutaminase | GLS | 0 | 0.32 | 0.04 | 0.23 | 0.06 | 0.1 |  |  |  |  | 0.03 | 0.31 | 0.01 | 0.25 | 0.04 | 0.35 |
|  | growth factor, augmenter of liver regeneration | GFER | 0.04 | 0.38 | 0.01 | 0.36 | 0.31 | 0.43 | 0 | 0.4 | 0 | 0.4 | 0.06 | 0.33 | 0.16 | 0.32 | 0.01 | 0.35 |
|  | hydroxysteroid 17-beta dehydrogenase 10 | HSD17B10 | 0.06 | 0.13 | 0.07 | 0.2 | 0.13 | 0.21 | 0.04 | 0.21 | -0.03 | 0.17 | 0.08 | 0.24 | 0.06 | 0.22 | 0.02 | 0.19 |
|  | Janus kinase 2 | JAK2 | 0.4 | 0.17 | 0.44 | 0.24 | 0.44 | 0.15 | 0.32 | 0.2 | 0.42 | 0.19 | 0.49 | 0.24 | 0.45 | 0.15 | 0.38 | 0.18 |
|  | MDM2 proto-oncogene | MDM2 | 0.49 | 0.36 | 0.44 | 0.22 | 0.46 | 0.21 | 0.61 | 0.11 | 0.41 | 0.18 | 0.57 | 0.28 | 0.56 | 0.29 | 0.55 | 0.34 |
|  | phosphatidylinositol-5-phosphate 4-kinase type 2 alpha | PIP4K2A | 0.03 | 0.39 | 0.1 | 0.4 | 0.07 | 0.21 |  |  | -0.01 | 0.17 | 0.07 | 0.38 | 0 | 0.37 | 0.06 | 0.38 |
|  | phospholipase A2 group VII | PLA2G7 | -0.01 | 0.38 | 0.01 | 0.25 | 0.15 | 0.35 |  |  | -0.01 | 0.35 | 0.09 | 0.2 | 0.14 | 0.24 | 0.03 | 0.27 |
|  | polo like kinase 1 | PLK1 | 0.03 | 0.31 | 0.05 | 0.26 | 0.09 | 0.05 | -0.02 | 0.21 | 0.01 | 0.07 | 0.06 | 0.33 | 0.04 | 0.31 | 0 | 0.36 |
|  | serine/threonine kinase 33 | STK33 | 0.45 | 0.13 | 0.34 | 0.24 | 0.49 | 0.15 | 0.5 | 0.2 | 0.41 | 0.11 | 0.41 | 0.21 | 0.37 | 0.26 | 0.33 | 0.29 |
|  | ubiquitin specific peptidase 1 | USP1 | -0.01 | 0.08 | 0.04 | 0.1 | 0.03 | 0.05 | 0.03 | 0.06 | -0.01 | 0.12 | 0.04 | 0.07 | 0.14 | 0.1 | -0.01 | 0.09 |
|  | YES proto-oncogene 1, Src family tyrosine kinase | YES1 | 0.39 | 0.44 | 0.34 | 0.42 | 0.43 | 0.44 | 0.26 | 0.39 | 0.32 | 0.43 | 0.33 | 0.53 | 0.41 | 0.45 | 0.08 | 0.38 |
| Epigenetic regulator | bromodomain adjacent to zinc finger domain 2B | BAZ2B | 0.24 | 0.36 | 0.1 | 0.34 | 0.31 | 0.37 | 0.07 | 0.21 | 0.01 | 0.37 | 0.1 | 0.3 | 0.2 | 0.28 | 0.08 | 0.31 |
|  | chromobox 1 | CBX1 | 0.16 | 0.12 | 0.14 | 0.2 | 0.2 | 0.19 | 0.17 | 0.21 | 0.1 | 0.11 | 0.17 | 0.14 | 0.15 | 0.17 | 0.12 | 0.15 |
|  | lysine demethylase 4A | KDM4A | 0.32 | 0.24 | 0.15 | 0.31 | 0.36 | 0.39 | 0.2 | 0.26 | 0.22 | 0.3 | 0.17 | 0.32 | 0.22 | 0.27 | 0.06 | 0.24 |
|  | lysine demethylase 4E | KDM4E | 0.24 | 0.48 | 0.05 | 0.43 | 0.31 | 0.43 | 0.09 | 0.42 | 0.01 | 0.36 | 0.08 | 0.44 | 0.19 | 0.45 | 0.06 | 0.41 |
|  | M-phase phosphoprotein 8 | MPHOSPH8 | 0.03 | 0.1 | 0.06 | 0.32 | 0.11 | 0.36 | 0.02 | 0.36 | 0 | 0.15 | 0.28 | 0.28 | 0.09 | 0.29 | 0.03 | 0.31 |
|  | protein arginine methyltransferase 1 | PRMT1 | 0.01 | 0.22 | 0.04 | 0.32 | 0 | 0.04 |  |  |  |  | 0.04 | 0.31 | 0.02 | 0.34 | 0.02 | 0.41 |
|  | sirtuin 5 | SIRT5 |  |  | 0.02 | 0.19 |  |  |  |  |  |  | 0.02 | 0.13 | 0 | 0.11 | 0 | 0.1 |
|  | survival of motor neuron 2, centromeric | SMN2 |  |  | 0.02 | 0.16 | 0.06 | 0.07 |  |  |  |  | 0.07 | 0.17 | 0.07 | 0.15 | -0.01 | 0.19 |

Supplementary Table 1 (continued): Mean MCCs of models (mean per condition).

|  |  | Cell line | A375 | | A549 | | HA1E | | HCC515 | | HT29 | | MCF7 | | PC3 | | VCAP | |
| --- | --- | --- | --- | --- | --- | --- | --- | --- | --- | --- | --- | --- | --- | --- | --- | --- | --- | --- |
| Target class | Target name | Target / Descriptor | GES | Morgan FP | GES | Morgan FP | GES | Morgan FP | GES | Morgan FP | GES | Morgan FP | GES | Morgan FP | GES | Morgan FP | GES | Morgan FP |
| Ion channel | potassium voltage-gated channel H2 | KCNH2 | 0.48 | 0.66 | 0.32 | 0.63 | 0.36 | 0.6 | 0.32 | 0.55 | 0.5 | 0.61 | 0.32 | 0.67 | 0.32 | 0.62 | 0.37 | 0.62 |
| Membrane receptor | 5-hydroxytryptamine receptor 1A | HTR1A | 0.01 | 0.47 | 0.03 | 0.5 | 0.12 | 0.48 | 0.09 | 0.52 | 0.03 | 0.41 | 0.16 | 0.51 | 0.07 | 0.46 | 0.11 | 0.55 |
|  | cholinergic receptor muscarinic 1 | CHRM1 | 0.23 | 0.33 | 0.29 | 0.26 | 0.36 | 0.32 | 0.43 | 0.39 | 0.3 | 0.28 | 0.2 | 0.28 | 0.24 | 0.28 | 0.29 | 0.28 |
|  | cholinergic receptor muscarinic 4 | CHRM4 | 0.27 | 0.35 | 0.33 | 0.33 | 0.31 | 0.33 | 0.41 | 0.36 | 0.22 | 0.26 | 0.28 | 0.24 | 0.23 | 0.26 | 0.26 | 0.25 |
|  | cholinergic receptor muscarinic 5 | CHRM5 | 0.2 | 0.38 | 0.31 | 0.33 | 0.29 | 0.34 | 0.45 | 0.42 | 0.23 | 0.34 | 0.27 | 0.27 | 0.21 | 0.25 | 0.23 | 0.25 |
|  | dopamine receptor D1 | DRD1 | 0.3 | 0.32 | 0.23 | 0.34 | 0.25 | 0.33 | 0.23 | 0.34 | 0.25 | 0.37 | 0.21 | 0.32 | 0.23 | 0.31 | 0.19 | 0.33 |
|  | dopamine receptor D2 | DRD2 | 0.25 | 0.4 | 0.26 | 0.45 | 0.25 | 0.48 | 0.24 | 0.47 | 0.23 | 0.4 | 0.28 | 0.43 | 0.24 | 0.41 | 0.31 | 0.46 |
|  | dopamine receptor D3 | DRD3 | 0.21 | 0.26 | 0.15 | 0.28 | 0.12 | 0.32 | 0.19 | 0.35 | 0.17 | 0.26 | 0.22 | 0.28 | 0.12 | 0.29 | 0.18 | 0.31 |
|  | neuropeptide S receptor 1 | NPSR1 |  |  | 0.22 | 0.38 | -0.01 | 0.12 |  |  |  |  | 0.26 | 0.39 | 0.16 | 0.35 | 0.19 | 0.34 |
|  | opioid receptor kappa 1 | OPRK1 | 0.01 | 0.27 | 0.06 | 0.33 | -0.01 | 0.27 |  |  | 0.16 | 0.34 | 0.15 | 0.24 | 0.09 | 0.27 | 0.09 | 0.35 |
|  | thyroid stimulating hormone receptor | TSHR | 0.02 | 0.26 | 0 | 0.08 | 0.15 | 0.03 | 0.22 | 0 |  |  | 0.1 | 0.13 | 0.11 | 0.12 | 0.14 | 0.17 |
|  | TNF receptor superfamily member 10b | TNFRSF10B |  |  | 0.38 | 0.18 | 0.12 | 0.05 |  |  | 0.42 | 0.15 | 0.3 | 0.17 | 0.44 | 0.12 | 0.34 | 0.11 |
| Other cytosolic protein | heat shock protein 90 alpha A1 | HSP90AA1 | 0.05 | 0.38 | 0.01 | 0.37 | 0.32 | 0.45 |  |  | 0.13 | 0.41 | 0.14 | 0.27 | 0.22 | 0.28 | 0.01 | 0.28 |
|  | heat shock protein family B1 | HSPB1 | 0.22 | 0.15 | 0.15 | 0.05 | 0.32 | 0.14 | 0.17 | 0.02 | -0.01 | 0.1 | 0.33 | 0.14 | 0.31 | 0.17 | 0.11 | 0.1 |
| Secreted protein | interleukin 1 beta | IL1B | 0.29 | 0.09 | 0.32 | 0.21 | 0.33 | 0.1 | 0.31 | 0.13 | 0.39 | 0.11 | 0.36 | 0.25 | 0.36 | 0.23 | 0.31 | 0.23 |
| Structural protein | tubulin beta class I | TUBB |  |  | 0.7 | 0.72 | 0.74 | 0.66 |  |  |  |  | 0.74 | 0.61 | 0.73 | 0.56 | 0.68 | 0.69 |
| Transcription factor | androgen receptor | AR | 0.04 | 0.25 | 0.19 | 0.48 | 0.15 | 0.41 | 0.11 | 0.48 | 0.02 | 0.24 | 0.28 | 0.49 | 0.14 | 0.42 | 0.33 | 0.45 |
|  | Jun proto-oncogene, AP-1 transcription factor subunit | JUN | 0.27 | 0.37 | 0.14 | 0.26 | 0.21 | 0.26 | 0.2 | 0.28 | 0.3 | 0.35 | 0.26 | 0.25 | 0.19 | 0.28 | 0.25 | 0.24 |
|  | melanogenesis associated transcription factor | MITF | 0.48 | 0.28 | 0.35 | 0.12 | 0.37 | 0.12 | 0.44 | 0.13 | 0.4 | 0.12 | 0.46 | 0.17 | 0.46 | 0.18 | 0.32 | 0.15 |
|  | nuclear factor kappa B1 | NFKB1 | 0.02 | 0.04 | 0.01 | 0.22 | 0.03 | 0 | 0 | 0.01 | 0.1 | 0 | 0 | 0.16 | 0.02 | 0.18 | 0.02 | 0.17 |
|  | nuclear receptor 3C1 | NR3C1 |  |  | 0.6 | 0.9 | 0.53 | 0.88 | 0.53 | 0.91 |  |  | 0.31 | 0.86 | 0.58 | 0.89 | 0.44 | 0.87 |
|  | nuclear receptor 5A1 | NR5A1 | 0.13 | 0.07 | 0.37 | 0.17 | 0.33 | 0.18 |  |  |  |  | 0.37 | 0.25 | 0.47 | 0.28 | 0.36 | 0.24 |
|  | tumor protein p53 | TP53 | 0.38 | 0.11 | 0.21 | 0.07 | 0.26 | 0.08 | 0.38 | 0.12 | 0.24 | 0.15 | 0.33 | 0.11 | 0.32 | 0.1 | 0.2 | 0.08 |
|  | vitamin D receptor | VDR | 0.01 | 0.12 | 0.01 | 0.11 | 0.05 | 0.11 | 0.05 | 0.08 | 0.05 | 0.08 | 0.12 | 0.13 | 0.09 | 0.11 | 0.06 | 0.12 |
| Transporter | abhydrolase domain containing 5 | ABHD5 | 0.04 | 0.12 | 0.01 | 0.26 |  |  |  |  |  |  | 0.09 | 0.29 | 0.08 | 0.26 | 0.08 | 0.34 |
|  | solute carrier family 6 member 3 | SLC6A3 | 0.32 | 0.31 | 0.33 | 0.27 | 0.36 | 0.26 | 0.38 | 0.25 | 0.26 | 0.27 | 0.33 | 0.23 | 0.34 | 0.24 | 0.33 | 0.3 |
| Unclassified protein | ataxin 2 | ATXN2 | 0.55 | 0.03 | 0.4 | 0.23 | 0.45 | 0.05 | 0.43 | 0.08 | 0.45 | 0.16 | 0.39 | 0.19 | 0.4 | 0.17 | 0.38 | 0.2 |
|  | ATPase family, AAA domain containing 5 | ATAD5 | 0.18 | 0.09 | 0.08 | 0.19 | 0.21 | 0.13 | 0.17 | 0.17 | 0.1 | 0.13 | 0.16 | 0.16 | 0.24 | 0.17 | 0.04 | 0.19 |
|  | endothelial PAS domain protein 1 | EPAS1 |  |  | 0.23 | 0.26 |  |  |  |  |  |  | 0.33 | 0.39 | 0.4 | 0.32 | 0.34 | 0.4 |
|  | geminin, DNA replication inhibitor | GMNN | 0.35 | 0.15 | 0.41 | 0.12 | 0.35 | 0.07 | 0.3 | 0.24 | 0.37 | 0.08 | 0.41 | 0.16 | 0.43 | 0.13 | 0.39 | 0.16 |
|  | MLLT3, super elongation complex subunit | MLLT3 |  |  | 0.01 | 0.04 |  |  |  |  |  |  | 0.07 | 0.04 | 0.02 | 0.15 | 0.01 | 0.21 |
|  | MYC proto-oncogene, bHLH transcription factor | MYC |  |  | 0.48 | 0.23 |  |  |  |  |  |  | 0.45 | 0.23 | 0.33 | 0.24 | 0.47 | 0.21 |
|  | nuclear factor, erythroid 2 like 2 | NFE2L2 | 0.13 | 0.02 | 0.14 | 0.16 | 0.11 | 0.13 | 0.19 | 0.15 | 0.16 | 0.05 | 0.1 | 0.17 | 0.18 | 0.15 | 0.15 | 0.16 |
|  | nucleotide binding oligomerization domain containing 1 | NOD1 |  |  | 0.22 | 0.37 |  |  |  |  | 0.23 | 0.16 | 0.33 | 0.32 | 0.29 | 0.31 | 0.21 | 0.42 |
|  | nucleotide binding oligomerization domain containing 2 | NOD2 | 0 | 0.08 | 0.06 | 0.19 | 0.11 | 0.09 |  |  | 0.08 | 0.08 | 0.15 | 0.28 | 0.18 | 0.26 | 0.13 | 0.22 |
|  | RAD52 homolog, DNA repair protein | RAD52 |  |  | 0.1 | 0.1 |  |  |  |  |  |  | 0.06 | 0.09 | 0.05 | 0.15 | 0.04 | 0.21 |
|  | TAR DNA binding protein | TARDBP |  |  | 0.01 | 0.14 | 0.01 | 0.05 |  |  |  |  | 0.05 | 0.09 | 0.07 | 0.06 | -0.01 | 0.11 |
